# Supplementary material for: Reliability and Discriminative Validity of Wearable Sensors for the Quantification of Upper Limb Movement Disorders in Individuals with Dyskinetic Cerebral Palsy
Source: Sensors (Basel). 2023 Feb 1;23(3):1574. doi: 10.3390/s23031574 (PMC9921560; doi:10.3390/s23031574)
Supplement: Supplementary file 1 [file sensors-23-01574-s001.zip › Table_S1.pdf]

| Participant | Age | SEX | MACS level | L/R handed | Measured side |
|---|---|---|---|---|---|
| CP1 | 12.2y | M | 3 | R | L |
| CP2 | 23.9y | F | 1 | R | L |
| CP3 | 23.9y | F | 1 | R | R |
| CP4 | 18.5y | F | 3 | L | L |
| CP5 | 25.4y | M | 3 | L | R |
| CP6 | 16.2y | M | 2 | R | L |
| CP7 | 16.2y | M | 2 | R | R |
| CP8 | 13.5y | M | 2 | L | R |
| CP9 | 19.4y | F | 2 | R | L |
| CP10 | 16.9y | M | 3 | R | L |
| CP11 | 16.9y | M | 3 | R | R |
| CP12 | 11.9y | M | 2 | R | L |
| CP13 | 11.9y | M | 2 | R | R |
| CP14 | 13.6y | F | 3 | R | L |
| CP15 | 21.6y | F | 3 | R | L |
| CP16 | 8.6y | M | 2 | L | R |
| CP17 | 22.6y | M | 2 | L | R |
| CP18 | 9.6y | M | 2 | R | L |
| CP19 | 5.0y | M | 3 | L | R |
| CP20 | 19.2y | F | 3 | R | L |
| CP21 | 21.2y | M | 2 | R | L |
| CP22 | 20.2y | M | 2 | L | R |
| **Mean** | **16.7y** | | | | |
| | | | | | |
| TD1 | 9.4y | M | / | R | L |
| TD2 | 19.9y | M | / | R | L |
| TD3 | 19.1y | F | / | R | L |
| TD4 | 19.1y | F | / | R | L |
| TD5 | 14.1y | F | / | L | R |
| TD6 | 17.0y | F | / | R | L |
| TD7 | 18.2y | M | / | R | L |
| TD8 | 21.2y | F | / | R | L |
| TD9 | 21.5y | F | / | R | L |
| TD10 | 21.4y | F | / | R | L |
| TD11 | 22.2y | F | / | L | R |
| TD12 | 13.5y | M | / | L | R |
| TD13 | 19.5y | F | / | R | L |
| TD14 | 15.0y | M | / | R | L |
| TD15 | 12.1y | M | / | R | L |
| TD16 | 21.0y | F | / | R | L |
| TD17 | 14.4y | F | / | L | R |
| TD18 | 12.9y | F | / | R | L |
| TD19 | 15.1y | M | / | R | L |
| TD20 | 16.9y | M | / | R | L |
| **Mean** | **17.2y** | | | | |

Table S1. Participant characteristics. CP = cerebral palsy; TD = typically developing; MACS = Manual Ability Classification System.
