# Supplementary material for: Reliability and Discriminative Validity of Wearable Sensors for the Quantification of Upper Limb Movement Disorders in Individuals with Dyskinetic Cerebral Palsy
Source: Sensors (Basel). 2023 Feb 1;23(3):1574. doi: 10.3390/s23031574 (PMC9921560; doi:10.3390/s23031574)
Supplement: Supplementary file 1 [file sensors-23-01574-s001.zip › Table_S2.pdf]

	WITHIN SESSION RELIABILITY						BETWEEN SESSION RELIABILITY					
	Hand		Forearm		Upper arm		Hand		Forearm		Upper arm	
	ICC LONG	ICC SHORT	ICC LONG	ICC SHORT	ICC LONG	ICC SHORT	ICC LONG	ICC SHORT	ICC LONG	ICC SHORT	ICC LONG	ICC SHORT
**REACH FORWARD**												
Max Jerk	0.70 [0.43-0.88]	0.41 [0.24-0.64]	0.76 [0.55-0.90]	0.65 [0.49-0.80]	0.38 [0.08-0.68]	0.77 [0.64-0.88]	0.85 [0.54-0.95]	0.89 [0.67-0.97]	0.89 [0.71-0.96]	0.79 [0.44-0.92]	0.96 [0.88-0.99]	0.77 [036-0.92]
Max Ang Jerk	0.51 [0.19-0.88]	0.30 [0.15-0.54]	0.69 [0.44-0.86]	0.45 [0.28-0.65]	0.71 [0.47-0.87]	0.50 [0.34-0.69]	0.60 [0.00-0.87]	0.34 [-0.68-0.85]	0.71 [0.19-0.90]	0.69 [0.17-0.89]	0.98 [0.96-0.99]	0.72 [0.21-0.90]
Mean Acc	0.90 [0.78-0.96]	0.79 [0.65-0.90]	0.80 [0.61-0.92]	0.86 [0.77-0.93]	0.80 [0.60-0.92]	0.93 [0.87-0.96]	0.68 [0.05-0.90]	0.66 [0.33-0.94]	0.80 [0.45-0.93]	0.83 [0.54-0.94]	0.83 [0.53-0.94]	0.83 [0.52-0.94]
Max Acc	0.76 [0.53-0.91]	0.69 [0.53-0.84]	0.84 [0.67-0.94]	0.76 [0.63-0.87]	0.50 [0.21-0.76]	0.91 [0.85-0.96]	0.77 [0.30-0.92]	0.82 [0.44-0.94]	0.86 [0.62-0.95]	0.87 [0.64-0.95]	0.92 [0.80-0.97]	0.84 [0.57-0.94]
Mean Gyr	0.89 [0.75-0.96]	0.76 [0.62-0.88]	0.87 [0.73-0.95]	0.85 [0.75-0.92]	0.87 [0.74-0.95]	0.95 [0.92-0.98]	0.77 [0.29-0.92]	0.86 [0.53-0.96]	0.91 [0.72-0.97]	0.84 [0.59-0.94]	0.91 [0.69-0.97]	0.93 [0.81-0.98]
Max Gyr	0.80 [0.60-0.93]	0.73 [0.58-0.87]	0.88 [0.76-0.95]	0.72 [0.57-0.85]	0.77 [0.55-0.90]	0.94 [0.90-0.97]	0.74 [0.24-0.91]	0.89 [0.68-0.97]	0.70 [0.19-0.89]	0.91 [0.73-0.97]	0.95 [0.87-0.98]	0.95 [0.86-0.98]
RMS Acc	0.91 [0.80-0.97]	0.79 [0.66-0.90]	0.83 [0.67-0.93]	0.87 [0.78-0.93]	0.76 [0.54-0.90]	0.93 [0.88-0.97]	0.71 [0.13-0.91]	0.83 [0.45-0.95]	0.83 [0.52-0.94]	0.84 [0.57-0.94]	0.87 [0.64-0.95]	0.84 [0.56-0.94]
RMS Gyr	0.91 [0.78-0.97]	0.78 [0.64-0.89]	0.90 [0.80-0.96]	0.83 [0.73-0.91]	0.87 [0.74-0.95]	0.96 [0.92-0.98]	0.81 [0.42-0.94]	0.88 [0.60-0.96]	0.91 [0.74-0.97]	0.87 [0.65-0.95]	0.94 [0.80-0.97]	0.94 [0.84-0.98]
SE Acc	0.80 [0.59-0.92]	0.28 [0.14-0.49]	0.85 [0.70-0.94]	0.32 [0.17-0.53]	0.73 [0.49-0.89]	0.38 [0.23-0.59]	0.88 [0.62-0.96]	0.65 [-0.02-0.89]	0.94 [0.83-0.98]	0.78 [0.39-0.92]	0.86 [0.61-0.95]	0.79 [0.40-0.92]
SE Gyr	0.72 [0.46-0.89]	0.41 [0.24-0.62]	0.75 [0.52-0.90]	0.38 [0.22-0.59]	0.65 [0.38-0.84]	0.33 [0.18-0.54]	0.89 [0.50-0.97]	0.79 [0.37-0.93]	0.85 [0.56-0.95]	0.90 [0.73-0.96]	0.84 [0.55-0.94]	0.15 [-1.5-0.70]
**REACH & GRASP VERRTICAL**												
Max Jerk	0.87 [0.74-0.94]	0.42 [0.25-0.65]	0.71 [0.49-0.86]	0.32 [0.17-0.54]	0.68 [0.45-0.85]	0.49 [0.33-0.69]	0.55 [-0.55-0.86]	0.74 [0.19-0.92]	0.66 [0.00-0.88]	0.87 [0.65-0.95]	0.60 [-0.2-0.86]	0.39 [-0.83-0.79]
Max Ang Jerk	0.84 [0.69-0.93]	0.25 [0.10-0.48]	0.72 [0.51-0.87]	0.27 [0.13-0.49]	0.66 [0.43-0.84]	0.39 [0.24-0.60]	0.69 [-0.02-0.91]	0.72 [0.12-0.92]	0.83 [0.50-0.94]	0.80 [0.50-0.92]	0.75 [0.27-0.92]	0.47 [-0.53-0.81]
Mean Acc	0.89 [0.79-0.95]	0.77 [0.63-0.89]	0.82 [0.66-0.92]	0.73 [0.59-0.85]	0.73 [0.52-0.87]	0.78 [0.66-0.89]	0.58 [-0.26-0.88]	0.67 [-0.15-0.90]	0.69 [-0.02-0.90]	0.90 [0.74-0.96]	0.67 [0.04-0.88]	0.47 [-0.36-0.81]
Max Acc	0.87 [0.75-0.94]	0.49 [0.32-0.71]	0.81 [0.65-0.92]	0.40 [0.24-0.61]	0.69 [0.47-0.86]	0.63 [0.47-0.79]	0.50 [-0.70-0.85]	0.60 [-0.13-0.88]	0.61 [-0.14-0.87]	0.92 [0.79-0.97]	0.79 [0.38-0.93]	0.43 [-0.60-0.80]
Mean Gyr	0.90 [0.80-0.96]	0.80 [0.68-0.91]	0.85 [0.72-0.93]	0.76 [0.62-0.87]	0.76 [0.57-0.89]	0.85 [0.75-0.92]	0.81 [0.16-0.95]	0.76 [0.24-0.93]	0.90 [0.36-0.97]	0.49 [-0.38-0.81]	0.77 [0.18-0.93]	0.77 [0.36-0.92]
Max Gyr	0.92 [0.84-0.97]	0.63 [0.44-0.80]	0.93 [0.85-0.97]	0.52 [0.35-0.71]	0.73 [0.52-0.87]	0.66 [0.51-0.81]	0.84 [0.49-0.95]	0.91 [0.69-0.97]	0.88 [0.66-0.96]	0.84 [0.58-0.94]	0.92 [0.78-0.97]	0.74 [0.25-0.91]
RMS Acc	0.91 [0.82-0.96]	0.75 [0.61-0.88]	0.81 [0.65-0.92]	0.71 [0.56-0.84]	0.77 [0.59-0.90]	0.77 [0.64-0.88]	0.57 [-0.19-0.86]	0.70 [0.00-0.91]	0.70 [0.13-0.90]	0.91 [0.78-0.97]	0.73 [0.23-0.91]	0.45 [-0.43-0.80]
RMS Gyr	0.95 [0.89-0.98]	0.80 [0.67-0.90]	0.89 [0.79-0.95]	0.71 [0.56-0.84]	0.81 [0.65-0.92]	0.84 [0.74-0.92]	0.85 [0.52-0.95]	0.83 [0.45-0.95]	0.92 [0.64-0.97]	0.64 [0.03-0.96]	0.81 [0.40-0.94]	0.80 [0.44-0.93]
SE Acc	0.82 [0.66-0.92]	0.29 [0.06-0.42]	0.42 [0.13-0.69]	0.18 [0.06-0.38]	0.33 [0.04-0.63]	0.32 [0.18-0.54]	0.35 [-0.63-0.78]	0.57 [-0.31-0.87]	0.60 [-0.07-0.86]	0.74 [0.20-0.91]	0.41 [-0.6-0.79]	0.04 [-1.23-0.64]
SE Gyr	0.77 [0.58-0.89]	0.26 [0.11-0.50]	0.42 [0.13-0.69]	0.36 [0.20-0.57]	0.42 [0.13-0.69]	0.40 [0.24-0.60]	0.42 [-0.32-0.80]	0.47 [-0.45-0.83]	0.76 [0.14-0.92]	0.87 [0.65-0.95]	0.62 [-0.04-0.86]	0.26 [-1.33-0.75]
**REACH SIDEWAYS**												
Max Jerk	0.86 [0.70-0.94]	0.47 [0.29-0.69]	0.79 [0.56-0.92]	0.48 [0.32-0.68]	0.50 [0.18-0.77]	0.68 [0.53-0.83]	0.89 [0.31-0.97]	0.82 [0.41-0.94]	0.72 [0.10-0.91]	0.79 [0.44-0.92]	0.83 [0.46-0.94]	0.71 [0.21-0.90]
Max Ang Jerk	0.80 [0.60-0.92]	0.35 [0.18-0.59]	0.75 [0.53-0.90]	0.37 [0.21-0.58]	0.42 [0.10-0.72]	0.46 [0.29-0.66]	0.86 [0.51-0.96]	0.64 [-0.18-0.89]	0.85 [0.52-0.95]	0.69 [0.17-0.89]	0.79 [0.33-0.93]	0.73 [0.25-0.90]
Mean Acc	0.94 [0.87-0.98]	0.83 [0.72-0.91]	0.90 [0.78-0.96]	0.78 [0.65-0.88]	0.87 [0.72-0.95]	0.73 [0.59-0.86]	0.58 [-0.25-0.88]	0.74 [-0.10-0.93]	0.59 [-1.5-0.87]	0.83 [0.54-0.87]	0.69 [0.09-0.90]	0.67 [0.15-0.88]
Max Acc	0.90 [0.79-0.96]	0.69 [0.53-0.84]	0.83 [0.63-0.93]	0.67 [0.51-0.82]	0.71 [0.46-0.88]	0.65 [0.49-0.80]	0.85 [0.31-0.96]	0.65 [-0.03-0.89]	0.70 [0.01-0.91]	0.87 [0.64-0.95]	0.84 [0.51-0.95]	0.60 [-0.05-0.85]
Mean Gyr	0.95 [0.89-0.98]	0.81 [0.69-0.91]	0.86 [0.71-0.95]	0.78 [0.65-0.88]	0.86 [0.71-0.95]	0.80 [0.69-0.90]	0.67 [-0.12-0.91]	0.68 [-0.13-0.90]	0.72 [0.19-0.91]	0.84 [0.59-0.94]	0.74 [0.22-0.91]	0.67 [0.12-0.88]
Max Gyr	0.89 [0.77-0.96]	0.69 [0.53-0.84]	0.83 [0.66-0.93]	0.68 [0.53-0.82]	0.63 [0.35-0.84]	0.72 [0.57-0.85]	0.77 [0.21-0.94]	0.77 [0.15-0.93]	0.87 [0.61-0.96]	0.91 [0.73-0.97]	0.90 [0.70-0.97]	0.82 [0.51-0.93]
RMS Acc	0.94 [0.87-0.98]	0.82 [0.71-0.92]	0.89 [0.77-0.96]	0.77 [0.65-0.88]	0.86 [0.71-0.95]	0.74 [0.60-0.84]	0.62 [-0.21-0.90]	0.77 [-0.06-0.94]	0.55 [-0.35-0.85]	0.84 [0.57-0.94]	0.71 [0.14-0.91]	0.66 [0.11-0.87]
RMS Gyr	0.96 [0.91-0.99]	0.83 [0.72-0.92]	0.89 [0.76-0.96]	0.77 [0.65-0.88]	0.89 [0.75-0.96]	0.82 [0.71-0.91]	0.75 [0.00-0.93]	0.73 [-0.06-0.92]	0.75 [0.26-0.92]	0.87 [0.65-0.95]	0.79 [0.38-0.93]	0.70 [0.21-0.89]
SE Acc	0.92 [0.83-0.97]	0.29 [0.13-0.52]	0.78 [0.57-0.91]	0.47 [0.30-0.67]	0.73 [0.49-0.89]	0.40 [0.24-0.61]	0.93 [0.72-0.98]	0.26 [-1.27-0.76]	0.90 [0.68-0.97]	0.78 [0.39-0.92]	0.83 [0.44-0.95]	0.69 [0.12-0.89]
SE Gyr	0.89 [0.76-0.96]	0.39 [0.22-0.62]	0.69 [0.43-0.87]	0.50 [0.34-0.70]	0.71 [0.45-0.88]	0.59 [0.43-0.76]	0.79 [0.29-0.94]	0.55 [-0.26-0.85]	0.88 [0.60-0.96]	0.90 [0.73-0.96]	0.86 [0.55-0.95]	0.75 [0.30-0.91]

Table S2. Within-and between -session intra-class correlation coefficients (ICC) and confidence intervals. Acc= acceleration; ang vel = angular velocity; SE = sample entropy.
