# Supplementary material for: Reliability and Discriminative Validity of Wearable Sensors for the Quantification of Upper Limb Movement Disorders in Individuals with Dyskinetic Cerebral Palsy
Source: Sensors (Basel). 2023 Feb 1;23(3):1574. doi: 10.3390/s23031574 (PMC9921560; doi:10.3390/s23031574)
Supplement: Supplementary file 1 [file sensors-23-01574-s001.zip › Table_S3.pdf]

	Hand		Forearm		Upper arm	
	SEM	MEAN DIFFERENCE	SEM	MEAN DIFFERENCE	SEM	MEAN DIFFERENCE
**REACH FORWARD**						
Max Jerk (m/s^3)	50.62	355.82	47.36	288.59	21.45	79.97
Max Ang Jerk (rad/s^2)	7849.15	1968.83	5331.42	1740.59	3190.64	1009.18
Mean Acc (m/s^2)	0.71	0.06	0.53	0.17	0.29	0.27
Max Acc (m/s^2)	2.36	15.58	1.69	13.10	0.65	4.87
Mean Gyr (rad/s)	0.40	0.19	0.30	0.20	0.29	0.06
Max Gyr (rad/s)	1.14	3.18	0.82	2.59	0.35	1.02
RMS Gyr (rad/s)	0.35	0.31	0.26	0.25	0.26	0.01
RMS Acc (m/s^2)	0.59	0.70	0.50	0.69	0.27	0.45
SE Acc	0.17	0.05	0.17	0.03	0.21	0.04
SE Gyr	0.17	0.03	0.16	0.01	0.12	0.05
**REACH & GRASP VERTICAL**						
Max Jerk (m/s^3)	50.62	355.82	47.36	288.59	15.31	97.67
Max Ang Jerk (rad/s^2)	7849.15	1968.83	5331.42	1740.59	2445.10	1478.28
Mean Acc (m/s^2)	0.71	0.06	0.53	0.17	0.20	0.25
Max Acc (m/s^2)	2.36	15.58	1.69	13.10	0.51	5.78
Mean Gyr (rad/s)	0.40	0.19	0.30	0.20	0.23	0.03
Max Gyr (rad/s)	1.14	3.18	0.82	2.59	0.25	1.87
RMS Gyr (rad/s)	0.35	0.31	0.26	0.25	0.20	0.03
RMS Acc (m/s^2)	0.59	0.70	0.50	0.69	0.18	0.43
SE Acc	0.17	0.05	0.17	0.03	0.19	0.07
SE Gyr	0.17	0.03	0.16	0.01	0.10	0.02
**REACH SIDEWAYS**						
Max Jerk (m/s^3)	59.18	332.91	51.84	301.28	30.13	107.23
Max Ang Jerk (rad/s^2)	8649.15	1968.37	7076.13	2144.20	5776.75	1543.35
Mean Acc (m/s^2)	0.80	0.16	0.60	0.02	0.24	0.36
Max Acc (m/s^2)	2.18	15.22	1.64	14.89	0.83	6.61
Mean Gyr (rad/s)	0.45	0.05	0.41	0.07	0.28	0.01
Max Gyr (rad/s)	0.71	3.18	0.60	2.49	1.08	1.63
RMS Gyr (rad/s)	0.38	0.11	0.35	0.07	0.25	0.04
RMS Acc (m/s^2)	0.63	0.42	0.54	0.59	0.23	0.59
SE Acc	0.15	0.06	0.15	0.06	0.18	0.07
SE Gyr	0.13	0.00	0.10	0.03	0.11	0.04

Table S3. Between-session standard error of measurement (SEM) and mean difference between the DCP and TD group. Acc= acceleration; ang vel = angular velocity; SE = sample entropy.
