# Supplementary figures and images for: Reliability and Discriminative Validity of Wearable Sensors for the Quantification of Upper Limb Movement Disorders in Individuals with Dyskinetic Cerebral Palsy
Source: Sensors (Basel). 2023 Feb 1;23(3):1574. doi: 10.3390/s23031574 (PMC9921560; doi:10.3390/s23031574)

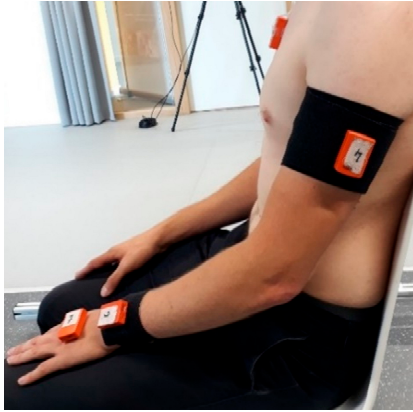

Figure S1. Sensor placements

Supplement: Supplementary file 1 [file sensors-23-01574-s001.zip › Figure_S1.pdf]

### Reach forwards

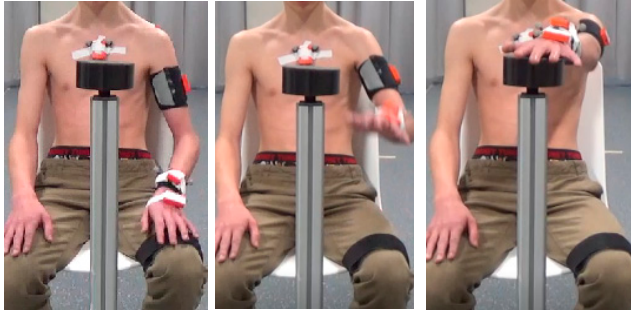

### Reach and grasp vertical

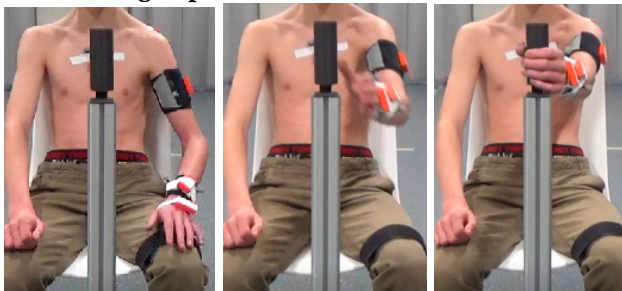

### Reach sideways

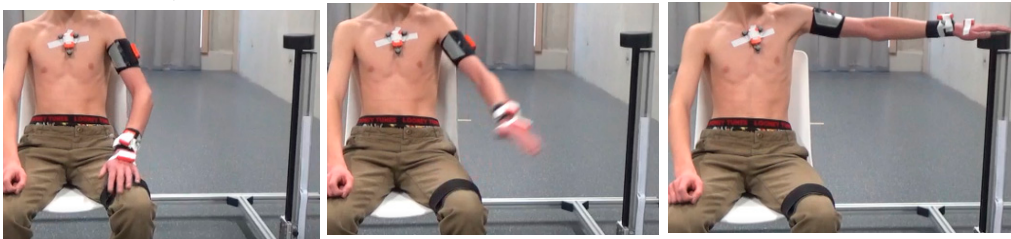

Figure S2. Task illustration

Supplement: Supplementary file 1 [file sensors-23-01574-s001.zip › Figure_S2.pdf]
